# Hybrid Carcinoma of the Larynx: A Case Report (Adenoid Cystic and Adenocarcinoma) and Review of the Literature

**DOI:** 10.1155/2013/385405

**Published:** 2013-04-04

**Authors:** Ilias Karasmanis, John K. Goudakos, Iosif Vital, Thomas Zarampoukas, Victor Vital, Konstantinos Markou

**Affiliations:** ^1^Department of Otorhinolaryngology, Head and Neck Surgery, Ippokratio Hospital, Konstantinoupoleos 49, 54642 Thessaloniki, Greece; ^2^1st Department of Otorhinolaryngology, Head and Neck Surgery, AHEPA University Hospital, Aristotle University of Thessaloniki, 1 Stilponos Kyriakidi Street, 54006 Thessaloniki, Greece; ^3^Department of Pathology, Medical School, Aristotle University of Thessaloniki, 54124 Thessaloniki, Greece

## Abstract

*Introduction*. The nonsquamous carcinomas of the larynx are considered rare with the majority of malignant tumors in this area, reaching the rate of 95%, to be squamous cell neoplasms. *Case Report*. The case refers to a 53-year-old man that presented with symptomatology of motor nerve disease. During the evaluation of the neurologic disease, a subglottic mass of the larynx was revealed accidentally in the imaging examination. Under general anesthesia, we performed direct laryngoscopy and biopsy of the mass. The histopathologic examination revealed a hybrid carcinoma coexistence of two different carcinomas, an adenoid cystic carcinoma and an adenocarcinoma, not otherwise specified with poor differentiation. Regarding the therapeutic plan, the mass was considered inoperable due to its expansion to trachea and the patient received radiotherapy. *Conclusions*. Both the adenocarcinoma and adenoid cystic carcinoma are extremely rare types of malignant tumors in the larynx. The special interest of the present case is the coexistence of these two rare tumors in the same region of the larynx, being a hybrid tumor of the salivary glands in the larynx, which is the second reported case, based on our systematic literature review.

## 1. Introduction

The cancer of larynx represents an important portion of clinical oncology, accounting for 30%–40% of the head and neck carcinomas and the 1%–2,5% of the whole human malignancies [1]. Predominant histological type remains the squamous cell carcinoma, representing 90%–95% of the new diagnosed cases [2]. Rare types of laryngeal carcinoma are neuroendocrine, adenocarcinoma, adenoid cystic, sarcomas (rhabdomyosarcoma, leiomyosarcoma, fibrosarcoma, and chondrosarcoma), parvicellular, primary lymphoma, plasmacytoma, and metastatic malignancies (kidney, lung, breast, and melanoma) [1, 2].

The adenoid cystic carcinomas are usually primary tumors of salivary glands, and their location in larynx is extremely rare, representing 0,1%–0,7% of the laryngeal carcinomas. The adenoid cystic tumors proceed from small ectopic submucosal salivary glands, presenting a slow development. An adenoid cystic carcinoma of the larynx is diagnosed in advanced stage as it causes no special symptoms and usually locates in supraglottic region [3, 4]. Laryngeal adenocarcinomas are also rare histologic type, accounting for approximately 0,5% of the tumors of larynx. The certain tumors, primary or secondary metastatic type, are considered exceptionally aggressive, presenting with a higher risk of local and regional metastases in comparison with other laryngeal malignancies [5, 6]. 

The aim of our study is the presentation of a case of hybrid carcinoma in the larynx, diagnosed and treated in our department and composed of adenoid cystic carcinoma and adenocarcinoma with poor differentiation and without special characters (not otherwise specified: NOS). The special interest of our patient is based on the infrequency of hybrid carcinomas of salivary glands in the region of larynx, with the systematic review of the literature to reveal that our patient would be the second reported case [7].

## 2. Case Report

**Figure 1:**
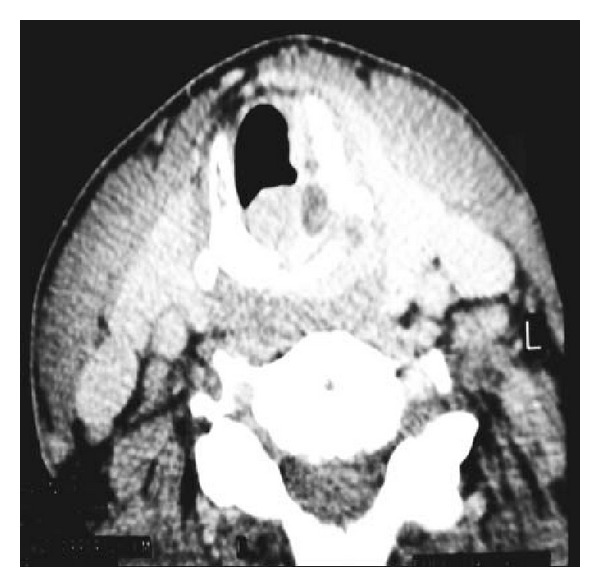
CT of neck. Invading mass of the larynx with subglottic and intralaryngeal extension.

**Figure 2:**
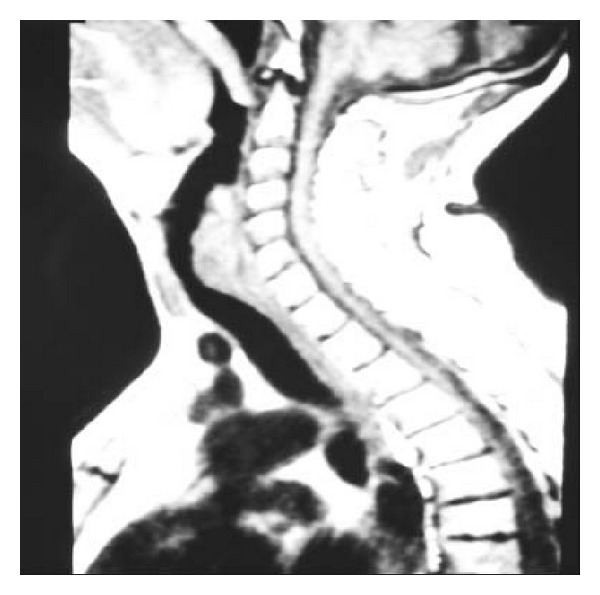
MRI of neck. Laryngeal mass, starting from the region of the left vocal cord and expanding in the trachea until the level of 4th thoracic vertebra.

Our patient, a 53-year-old man, presented to the neurology clinic of our hospital with symptomatology of motor nerve disease, reporting progressive weakness of both legs and arms during the last 4 months, especially in left side, difficulty to walk, muscle fasciculation, and cramps. During the neurologic diagnostic assessment, a subglottic mass of the larynx was found accidentally in the imaging examination of the head and neck. Particularly, the computed tomography (CT) and magnetic resonance imaging (MRI) of the neck revealed a mass with subglottic extension, starting from the region of the left vocal cord, invading the cricoid cartilage, and expanding in the trachea until the level of 4th thoracic vertebra (Figures 1 and 2). A smooth submucosal subglottic mass was emerged in endoscopic examination. In patient's history, symptoms related to this mass, such as dyspnoea, dysphagia, or hoarseness, were not reported. The patient was a heavy smoker, 20 cigarettes per day for 30 years, with positive cancer history as his father died due to laryngeal carcinoma.

**Figure 3:**
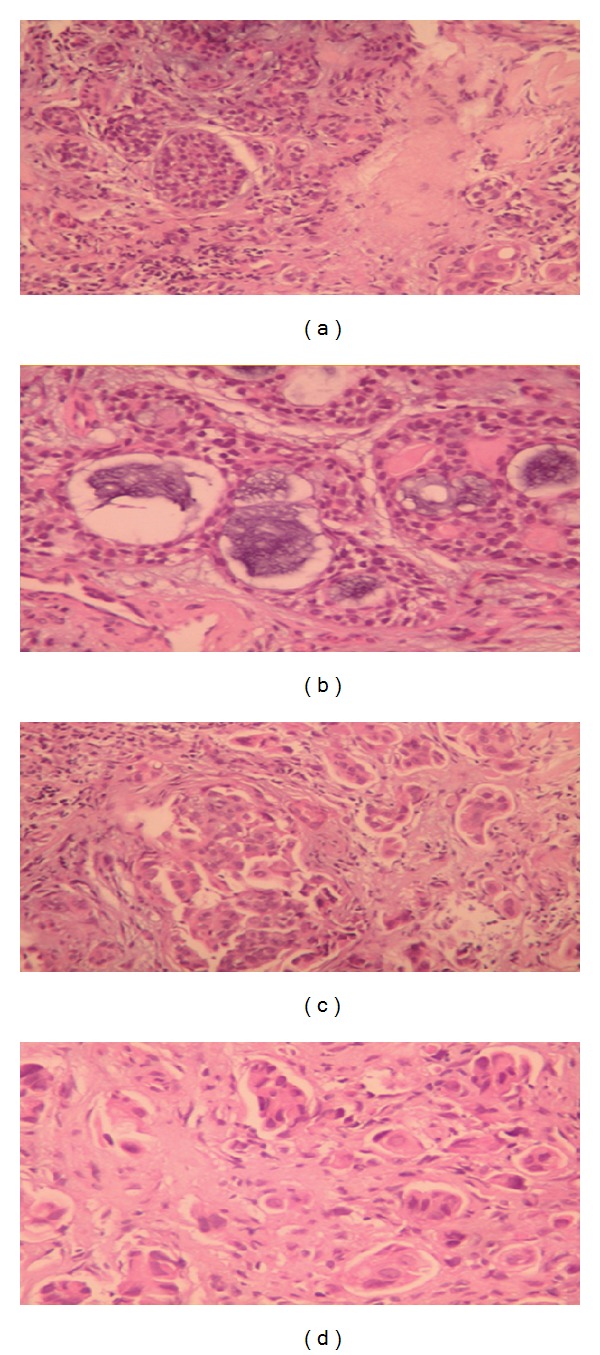
Histopathological examination. (a) and (b) The classical pattern of adenoid cystic carcinoma, with a mixture of cribriform and tubular pattern. (c) and (d) The glandular and solid growth pattern of the adenocarcinoma (NOS).

**Figure 4:**
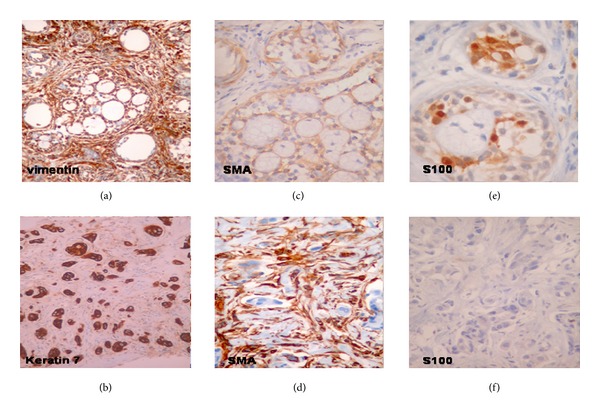
Immunochemistry. (a) Vimentin staining: the cells of the adenoid cystic carcinoma were positive. (b) Keratin 7 staining: the cells of both tumors were positive. (c) and (d) SMA staining: positive for the adenoid cystic carcinoma's cells and negative for the adenocarcinoma. (e) and (f) S100 staining: positive for the adenoid cystic carcinoma's cells and negative for the adenocarcinoma.

Under general anesthesia, direct laryngoscopy and biopsy from the mass were performed. The histopathologic (Figure 3) and immunohistochemical examination (Figure 4) revealed the coexistence of two different carcinomas, an adenoid cystic carcinoma and an adenocarcinoma—not otherwise specified (NOS) with poor differentiation. The adenoid cystic carcinoma presented the classical pattern, consisted of uniform appearance of small epithelial cells with mild atypia, minimum mitosis, and mixture of cribriform (mainly) and tubular pattern (Figures 3(a) and 3(b)). By contrast, the cells of adenocarcinoma NOS were larger with intense atypia and glandular or solid growth pattern (Figures 3(c) and 3(d)). In immunochemistry analysis, the cells of adenoid cystic carcinoma were positive for vimentin, *α*-SMA, and S-100 protein in contrast with the cells of adenocarcinoma that were negative (Figures 4(a)–4(f)). The cells of both tumors had positive staining for keratin 7 (Figure 4(b)). Histopathologically and immunochemically, there was a clear coexistence of two different types of carcinomas.

The completion of the imaging and endoscopic examination of the patient did not locate any other primary neoplasia in gastrointestinal and respiratory system. Regarding the therapeutic plan, the mass was considered inoperable due to its expansion to trachea and the patient received radiotherapy.

## 3. Discussion

The major interest of our case is the coexistence of two different types of carcinomas in the laryngeal tumor. It is a hybrid salivary gland carcinoma of adenocarcinoma and adenoid cystic neoplasm. Histopathologically, the most frequent laryngeal tumor is the squamous cell carcinoma with the nonepithelial neoplasms to represent less than 1%–3% of the whole tumors. Even rarer are the minor salivary gland tumors in larynx, such as adenocarcinoma and adenoid cystic, with their occurrence rate to be approximately 1% [2, 8]. 

The minor salivary gland tumors derive from minor submucosal glands, located mainly in subglottic region, in 60% of cases, while supraglottic or glottic cases have also been reported [3, 9]. The disease's progress is usually slow and the diagnosis in the most cases is made in advanced stages.

The symptomatology is informal and is characterized, based on tumor's location, by dyspnoea on exertion and hoarseness and in advanced stages by inspiratory whistle, dyspnoea at rest, and dysphagia. Characteristic example of the informal symptomatology of such laryngeal tumors is our case, as the patient denied any of the above-mentioned signs despite the large extension of the carcinoma. Clinically, the lesion spreads submucosally and is smooth and nonulcerated. Most cases are seen in the 5th or 6th decade of the patient's life, with a slight preponderance in females (3 : 2). Histopathologically, three types are distinguished into cribriform (the most frequent), tubular (with best prognosis), and solid (with the worst prognosis). 

Regarding the prognosis, the natural course of such tumors seems unforeseeable as they remain stable or grow slowly for many years, and, suddenly, they may become extremely aggressive [9–11]. The 5-year survival of adenoid cystic carcinoma in head and neck region is approximately 89% after the initial treatment. However, the 15-year survival is about 40%, indicant of the unforeseeable behavior of such tumors [12, 13]. In the larynx, the prognosis of adenoid cystic carcinoma is poor, as the published 5-year survival rates range from 15% to 33% [9, 10, 14]. The occurrence rate of lymph node metastasis varies between 5% and 15%, while the distal metastasis, due to hematogenous and perineural spread, occurs in 40% of cases with the lung to be the most frequent location. The treatment plan includes the surgical excision of the tumor and postoperative radiotherapy or/and chemotherapy. It should be noted that a long-term followup is needed with regular assessment of the respiratory system due to contingent natural of tumor [12, 15].

The salivary duct adenocarcinoma is a rare tumor, with its incidence to vary between 0.35% and 0.5% [5]. The tumor occurs mainly in males in the 6th or 7th decade of their life. While the clinical characteristics of adenocarcinoma are similar to adenoid cystic tumor, it is considered a neoplasm with higher grade of malignancy [5, 6]. At the time of adenocarcinoma's diagnosis, the rate of positive regional lymph nodes is 75% and the hazard of distal metastasis is about 46% [6]. As in treatment plan of adenocystic carcinomas, surgical excision with postoperative radiotherapy or/and chemotherapy is the therapy choice for adenocarcinomas. 

Seifert and Donath used the term hybrid tumor for salivary glands firstly in 1996 to report the coexistence of two different types of carcinomas, growing as a sole entity, microscopically and macroscopically [16]. The etiology theory remains unclear. The hybrid tumors are distinct lesions from collision tumors, which are characterized by the development of two different carcinomas in the same region. Hybrid tumors are extremely rare cases, accounting for the 0.1% of the whole salivary gland carcinomas [17–19]. Fewer than 30 cases of hybrid tumors have been described since now, with most of them occurring in parotid and the minor salivary glands of palate. Patients with hybrid carcinoma in submandibular gland, upper lip, antrum, lacrimal gland, and larynx have also been reported [7, 18, 19]. 

Due to the infrequency of hybrid tumors and the lack of published case series, our knowledge about the characteristics, the natural course, and the prognosis of such lesions remains limited. Most authors support that the biological behavior and the prognosis of a hybrid tumor are determined by the most aggressive of the coexisted carcinomas. For the same reason, the treatment is based on the therapeutic strategy for the most malignant of the coexisted tumors [18, 19]. 

## 4. Conclusions

The minor salivary gland tumors of the larynx are rare clinical entities, with the simultaneous coexistence of them in a hybrid carcinoma to be considered an extremely infrequent case. The systematic review of the literature revealed that our case is the second reported one of hybrid carcinoma (adenocarcinoma and adenoid cystic) of the larynx. The understanding of the clinical and histopathological characteristics and natural course of such tumors would lead to more effective therapeutic strategies.
